# The role of fatty acids in neurodegenerative diseases: mechanistic insights and therapeutic strategies

**DOI:** 10.1016/j.jlr.2025.100944

**Published:** 2025-11-12

**Authors:** Yufei Yang, Qingkun Wang, Zhaojun Wang, Yajing Wang, Beibei Liu, Youao Zhang, Xinyuan Mao, Haitao Sun

**Affiliations:** 1 Clinical Biobank Center, Guangdong Provincial Clinical Research Center for Laboratory Medicine, Department of Laboratory Medicine, Zhujiang Hospital and the Second Clinical Medical College, Southern Medical University, Guangzhou, China; 2 Neurosurgery Center, The National Key Clinical Specialty, The Engineering Technology Research Center of Education Ministry of China on Diagnosis and Treatment of Cerebrovascular Disease, Guangdong Provincial Key Laboratory on Brain Function Repair and Regeneration, The Neurosurgery Institute of Guangdong Province, Zhujiang Hospital Institute for Brain Science and Intelligence, Zhujiang Hospital, Southern Medical University, Guangzhou, China; 3 The First School of Clinical Medicine, Nanfang Hospital, Southern Medical University, Guangzhou, China; 4 Key Laboratory of Mental Health of the Ministry of Education, Guangdong-Hong Kong-Macao Greater Bay Area Center for Brain Science and Brain-Inspired Intelligence, Southern Medical University, Guangzhou, China

**Keywords:** fatty acids, neurodegenerative diseases, short-chain fatty acids, polyunsaturated fatty acids, neuroinflammation, oxidative stress, gut-brain axis, Alzheimer’s disease, Parkinson’s disease, amyotrophic lateral sclerosis

## Abstract

FAs play multifaceted roles in neurodegenerative diseases, including Alzheimer's disease, Parkinson's disease, and amyotrophic lateral sclerosis. This review systematically summarizes current understanding of FA metabolism and its diverse implications in neurodegenerative diseases pathology. Short-chain FAs, primarily generated by gut microbiota, regulate neuroinflammation, gut-brain communication, and blood-brain barrier integrity via epigenetic modifications and immune modulation. Medium-chain FAs exhibit therapeutic potential by improving energy metabolism and neuromuscular function, particularly in amyotrophic lateral sclerosis models. Long-chain PUFAs, notably DHA and EPA, contribute to neuronal membrane integrity, synaptic plasticity, and antioxidant defense, mitigating oxidative stress and neuroinflammation. Conversely, saturated and certain n-6 FAs may exacerbate neurodegeneration through proinflammatory and oxidative pathways. Emerging evidence highlights FA involvement in key pathological processes such as lipid peroxidation, mitochondrial dysfunction, ferroptosis, and blood-brain barrier disruption. Therapeutically, targeted supplementation, dietary modification, microbiome manipulation, and advanced nanotechnology-based delivery systems are promising strategies. Nevertheless, precise therapeutic efficacy depends critically on disease stage, dosage, genetic background, and individual metabolic context. Integrating personalized medicine with precision nutritional strategies and novel drug-delivery platforms offers promising avenues to translate FA-based interventions into clinical practice, potentially improving patient outcomes in the aging global population.

Neurodegenerative diseases (NDDs)—including Alzheimer's disease (AD), Parkinson's disease (PD), and amyotrophic lateral sclerosis (ALS)—are a group of complex disorders characterized by progressive neuronal degeneration. With the global population aging at an accelerating pace, the incidence of NDDs is rising markedly. Epidemiological data project that by 2050, the number of AD patients alone will exceed 100 million (1). This alarming trend not only severely compromises patients’ quality of life but also poses an unprecedented challenge to global public health systems. The lack of effective treatments, combined with the devastating impact of these diseases, underscores the urgent need to unravel their underlying pathogenic mechanisms.

Despite distinct clinical presentations, different NDDs share several key pathological features, including neuroinflammation, ferroptosis, oxidative stress, and BBB dysfunction (2). In recent years, advances in lipidomics have revealed a strong link between dysregulated FA metabolism and the onset and progression of NDDs (3, 4, 5). FAs are not only fundamental components of cell membranes and energy metabolism but also serve as crucial signaling molecules involved in diverse physiological processes. Notably, the role of FAs in disease is highly heterogeneous: different types of FAs may exert opposing biological effects, and their functions are dynamically modulated by disease stage, immune microenvironment, and genetic background (6, 7). However, most existing studies focus on isolated mechanisms and fail to comprehensively elucidate the dynamic interplay between FA metabolic networks and the multifaceted pathology of NDDs. Modern dietary shifts—particularly the widespread adoption of high-fat diets (HFDs) driven by industrialization—have led to imbalances in PUFA intake, which may exacerbate neurodegenerative processes by reshaping systemic lipid metabolism (8). Importantly, targeted supplementation of specific FAs has demonstrated promising therapeutic potential. For example, propionate, which belongs to short-chain fatty acids (SCFAs), enhances PINK1/PARKIN-mediated mitophagy and improves cognitive function in AD mouse models (9). These findings offer new avenues for the development of intervention strategies for NDDs.

This review aims to explore the relationship between various types of FAs and the pathogenesis of NDDs, with a focus on epigenetic modifications, BBB dysfunction, lipid peroxidation (LPO), mitochondrial dysfunction, ferroptosis, and neuroinflammation. It also summarizes recent advances linking FAs to the pathological features and clinical manifestations of NDDs, addressing critical gaps between mechanistic understanding and therapeutic application. These insights lay a theoretical foundation for the development of targeted fatty-acid-based interventions in NDDs.

## Classification and metabolism of FAs

### Classification of FAs

FAs are essential biomolecules composed of a carboxyl group attached to hydrocarbon chains of varying lengths. Based on carbon chain length, they are classified into SCFAs (C1-C6) such as acetate, propionate, and butyrate; medium-chain fatty acids (MCFAs, C7-C12) such as caprylic and capric acid; long-chain fatty acids (LCFAs, C13–C24) such as palmitic acid (PA), stearic acid, and DHA; and very long–chain fatty acids (VLCFAs, ≥C25) (10). FAs of different chain lengths serve distinct physiological roles. SCFAs are critical for maintaining intestinal barrier integrity, regulating inflammation, and modulating energy metabolism (11). MCFAs, due to their rapid absorption and metabolism, are widely used in clinical nutritional support (12). LCFAs and VLCFAs are key structural components of membrane lipids, with PUFAs—particularly DHA and EPA—being highly enriched in the brain and essential for neurodevelopment, synaptic plasticity, and neuroprotection (13). FAs are also categorized by the number of double bonds in their carbon chains: saturated fatty acids (SFAs), MUFAs, and PUFAs. PUFAs are further classified based on the position of the first double bond into n-3 (e.g., α-linolenic acid [ALA], DHA, EPA), n-6 (e.g., linoleic acid [LA], arachidonic acid [AA]), and n-9 (e.g., oleic acid [OA]) families. These PUFA subtypes exhibit distinct biological functions and have significant implications for human health (10).

### Sources of FAs

#### Dietary supplementation

Dietary intake is a key determinant in maintaining FA homeostasis in the human body. SCFAs are primarily produced by gut microbiota through the anaerobic fermentation of dietary fiber, with their synthesis efficiency jointly regulated by host dietary patterns and the composition of the intestinal microbiome (14, 15). Essential FAs, such as LA and α-linolenic acid (ALA), must be obtained from the diet. ALA is predominantly found in nuts, seeds, and plant oils (e.g., flaxseed oil, walnut oil) (16), while marine fish are rich sources of bioactive n-3 FAs, including DHA and EPA (17). In addition, coconut oil, palm kernel oil, and dairy products are rich in MCFAs, which can be rapidly absorbed without the need for bile emulsification. These properties make MCFAs particularly useful in clinical nutrition for patients with fat malabsorption (18).

#### FA biosynthesis

Endogenous FA synthesis primarily occurs in the liver and begins with acetyl-CoA, which is generated from pyruvate produced during glucose oxidation in the mitochondria and subsequently transported to the cytoplasm. Under the catalysis of acetyl-CoA carboxylase (ACC), acetyl-CoA is converted to malonyl-CoA, which is then used by FASN to sequentially produce PA—a 16-carbon SFA (10). PA can be further elongated by elongation of very long chain FA enzymes or desaturated by Δ4, Δ5, Δ6, and Δ9 desaturases to form unsaturated fatty acids (UFAs). However, humans lack Δ12 and Δ15 desaturases and are therefore unable to synthesize LA and ALA, making them essential FAs that must be obtained from the diet.

Although the liver is the principal site of FA synthesis, the central nervous system (CNS) also possesses significant lipogenic capacity, particularly given the restricted permeability of the blood-brain barrier (BBB) to exogenous lipids, which underscores the critical importance of local synthesis within the brain. Evidence indicates that astrocytes serve as central regulators of cerebral lipid metabolism, with the capacity to express crucial enzymes like ACC, FASN, and elongation of VLCFAs, thereby supporting the in situ biosynthesis of both saturated and UFAs (19). Such endogenous FAs are essential for the structural integrity of neuronal membranes, myelin formation, and modulation of synaptic plasticity. Notably, recent experimental evidence has further confirmed that FASN-mediated de novo lipogenesis in the brain is critical for neuronal differentiation and brain morphogenesis during embryonic and early developmental stages (20). Therefore, a comprehensive understanding of FA biosynthesis must consider CNS-specific lipid metabolic pathways and their essential functions in maintaining neural homeostasis.

### FA transport

FA transport plays a fundamental role in maintaining lipid homeostasis and neural function. Depending on their chain length, FAs utilize distinct transport mechanisms to reach and act within the CNS. SCFAs, mainly derived from microbial fermentation of dietary fiber, enter the circulation via monocarboxylate transporter 1 and cross the BBB through endothelial MCTs, contributing to cerebral energy metabolism and immune regulation (21, 22).

In contrast, LCFAs typically circulate in the periphery bound to albumin or incorporated into lipoproteins such as VLDL and LDL. These peripheral lipoproteins serve as major lipid transport carriers in systemic circulation but are generally restricted from entering the brain due to the tight regulation of the BBB. So LCFA uptake into the CNS relies on specialized membrane transporters, including CD36, FA transport proteins (FATP1/4), and plasma membrane-associated FA-binding protein (23). Notably, PUFAs, particularly DHA, rely on the MFSD2A transporter, which is highly expressed in brain microvascular endothelial cell, for efficient entry into the brain. Disruption of MFSD2A-mediated transport is associated with neurodevelopmental defects and cognitive decline, underscoring the importance of tightly regulated FA uptake in brain health (24).

Once within the CNS, astrocytes act as metabolic hubs, buffering excess FAs in lipid droplets (LDs) to prevent lipotoxicity while maintaining an energy reserve. Dysregulation of LD metabolism in astrocytes has been observed in AD and PD disease models, suggesting that aberrant lipid storage may contribute to neuroinflammation and neurodegeneration (25). Furthermore, astrocyte-neuron crosstalk facilitates intercellular lipid transport: astrocytes release FAs via apolipoprotein E (APOE)-associated particles to support neuronal membrane remodeling, synaptic plasticity, and energy demand—especially under stress or injury (26). Altogether, a dynamic FA transport network is essential for neural integrity, and its dysfunction may play a previously underappreciated role in the pathogenesis of neurodegenerative disorders. Elucidating these intercellular transport pathways may reveal new therapeutic avenues for modulating lipid metabolism in the diseased brain.

### FA oxidation

Although the brain accounts for only 2% of total body weight, it consumes approximately 20% of the body's energy (27). While glucose is the primary fuel under physiological conditions, alternative substrates such as FAs become critical during metabolic stress, including prolonged fasting or starvation (25). Under these circumstances, the brain can derive up to 20% of its energy from mitochondrial fatty acid oxidation (FAO), primarily occurring in astrocytes (28). However, it is worth noting that recent studies have found neuron-specific triglyceride lipases at hippocampus synaptic terminals, indicating that neurons can directly use LDs for FAO and energy, a discovery that changes the traditional perception that neurons rely only on glucose (29). FAs of varying chain lengths follow distinct catabolic pathways. SCFAs and MCFAs can diffuse into mitochondria and undergo rapid β-oxidation, while LCFAs require transport via the carnitine shuttle system, involving carnitine palmitoyltransferase (CPT) 1 and CPT2. VLCFAs, on the other hand, are predominantly oxidized in peroxisomes, underscoring the compartmentalized nature of FAO in the brain (30).

Glial cells, especially astrocytes and oligodendrocytes, play key roles in regulating cerebral lipid catabolism. Astrocytes can sequester neurotoxic FAs during heightened neuronal activity, store them in LDs, and subsequently metabolize them via β-oxidation to alleviate neuronal oxidative stress (26). In the myelin compartment, peroxisomal βOX in oligodendrocytes serves as an emergency energy source for axons. This mechanism has been validated in models of myelin loss induced by glucose transporter 1 deficiency (31).

Importantly, dysregulated FAO has been implicated in the pathogenesis of several NDDs. Genetic disruption of CPT2 in astrocytes leads to the accumulation of long-chain acylcarnitines in the hippocampus and cortex, contributing to mitochondrial dysfunction through the PPARα regulatory axis (32, 33). Furthermore, glucagon-like peptide-1 receptor activation in astrocytes promotes a metabolic shift from glucose metabolism to FAO, potentially heightening susceptibility to cognitive decline in AD models (34). Conversely, β-hydroxybutyrate, a FAO byproduct, has shown anti-inflammatory and neuroprotective properties by attenuating microglial activation and correcting metabolic imbalances in AD models (35). On the other hand, FAO is also a major source of reactive oxygen species (ROS), and excessive reliance on FAs as hydrogen donors can lead to oxidative stress and LPO in neurons, thereby contributing to the pathogenesis of AD, PD, and related disorders (36). These findings underscore the complexity of brain lipid metabolism and offer new perspectives on the mechanisms underlying NDDs.

## Mechanistic roles of FAs in NDDs

As key metabolic regulators in the CNS, FAs serve functions far beyond their traditional role as structural components of cell membranes. In NDDs, FAs participate in the regulation of diverse pathophysiological processes through intricate metabolic networks and have emerged as critical players in advancing our understanding of disease mechanisms. Recent studies reveal that dysregulated FA metabolism influences neurodegeneration through multiple mechanisms, including gut-brain axis signaling, maintenance of BBB integrity, modulation of oxidative stress and mitochondrial homeostasis, ferroptosis susceptibility, and neuroinflammation. Notably, different FA subtypes—such as SCFAs, PUFAs, and SFAs—exhibit pronounced “double-edged sword” effects in NDDs, exerting both neuroprotective and neurotoxic actions. These functional dichotomies display dynamic shifts across different stages of diseases such as AD, PD, and ALS. This section focuses on dissecting the mechanistic roles of FAs in these core pathological processes and exploring their potential therapeutic implications.

### SCFAs and the gut-brain axis

The gut-brain axis is a complex bidirectional communication network that regulates interactions between the CNS and the gastrointestinal (GI) tract. The CNS finely modulates GI motility and secretory functions through the autonomic nervous system and the hypothalamic-pituitary-adrenal axis (37), while the gut, in turn, exerts influence by producing bioactive metabolites, which affect neurodevelopment, inflammatory regulation, energy metabolism, and epigenetic modifications (38). In disorders such as AD and PD, mounting evidence suggests that gut microbiota dysbiosis and altered microbial metabolites can exacerbate cognitive dysfunction (39, 40, 41, 42, 43). Among these metabolites, SCFAs, as key products of microbial fermentation, have garnered considerable attention for their central role in mediating gut-brain communication (44).

In the CNS, SCFAs contribute to the epigenetic regulation of the nervous system primarily through histone modifications. Histone acetylation promotes gene transcription by adding acetyl groups to lysine residues on histone tails (45), a process reversible by histone deacetylases (HDACs) (45, 46). Aberrant expression of specific HDAC isoforms has been implicated in the pathogenesis of several CNS disorders. Notably, propionate and butyrate act as potent HDAC inhibitors within the CNS (47). In microbiota-depleted models, butyrate deficiency leads to increased HDAC4 activity in the hippocampus, which downregulates histone acetylation marks (H4K8ac, H4K12ac, H4K16ac), contributing to neuronal apoptosis (48). In the context of AD pathology, sodium butyrate supplementation selectively suppressed class I HDACs (HDAC1, HDAC2, HDAC3, HDAC8) in the hippocampal region, which significantly ameliorated memory formation and cognitive performance in a mouse model of AD (47), thereby underscoring the role of SCFAs in maintaining neuroplasticity via epigenetic reprogramming.

Beyond their epigenetic effects, gut-derived SCFAs modulate neuroinflammation by regulating glial cell development and cytokine release. According to studies, microbial metabolites such as acetate can promote metabolic maturation and reverse aberrant proliferation of microglia in AD model mice, while modulating their phagocytic activity to reduce hippocampal β-amyloid (Aβ) burden (49). Acetate suppresses the pro-inflammatory cascade in AD progression by inhibiting phosphorylation of NF-κB p65 in AD models (50). Meanwhile, gut-derived propionate modulates Aβ amyloidosis through reducing peripheral Th17 cells and interleukin (IL)-17 release (51). Additionally, peripheral supplementation of butyrate in AD mice effectively attenuates LPS-induced hippocampal IL-1β expression, with high-soluble-fiber diets exerting anti-inflammatory effects via elevated butyrate levels (52). However, this protective mechanism is vulnerable to disruption by SFAs (53). Concurrent bile acid dysregulation and microbiota imbalance may further impede the production of SCFA, creating a vicious cycle (54). It is worth noting that dietary interventions (such as n-3 PUFA and yeast β-glucan) can restore SCFA levels and improve cognitive impairment (55, 56).

SCFAs also integrate gut-brain signaling by modulating neurotransmitter metabolism. The dysregulation of the serotonergic system represents a critical component in the pathogenesis of AD. Degeneration of this system is not only closely associated with noncognitive symptoms such as depression and anxiety, frequently observed in early AD stages but also profoundly influences disease progression through direct modulation of Aβ pathology and cell survival pathways (57). Studies have demonstrated that serotonin can directly interact with Aβ42, effectively inhibiting the further aggregation of oligomers and fibrils as well as the formation of insoluble plaques by disrupting salt bridges and β-sheet structures within protofibrils (58). Notably, central and peripheral serotonin (5-hydroxytryptamine [5-HT]) levels are significantly regulated by SCFAs. Approximately 90% of 5-HT is synthesized by gut enterochromaffin cells, which signal the brainstem via vagal 5-HT3A receptors or enter systemic circulation through the serotonin transporter, affecting BBB function (59, 60). Acetic and butyric acids upregulate tryptophan hydroxylase in intestinal cells, promoting 5-HT synthesis, while butyrate further enhances serotonin transporter and 5-HTR expression to strengthen serotonergic signaling (61, 62). SCFAs also regulate other neurotransmitter pathways. Acetate from colonic fermentation increases hypothalamic glutamate levels (63), while SCFA mixtures enhance astrocyte–neuron metabolic coupling by modulating glutamine synthetase, thereby promoting the glutamate-glutamine shuttle and reducing oxidative stress in AD mice (64).

**Fig. 1:**
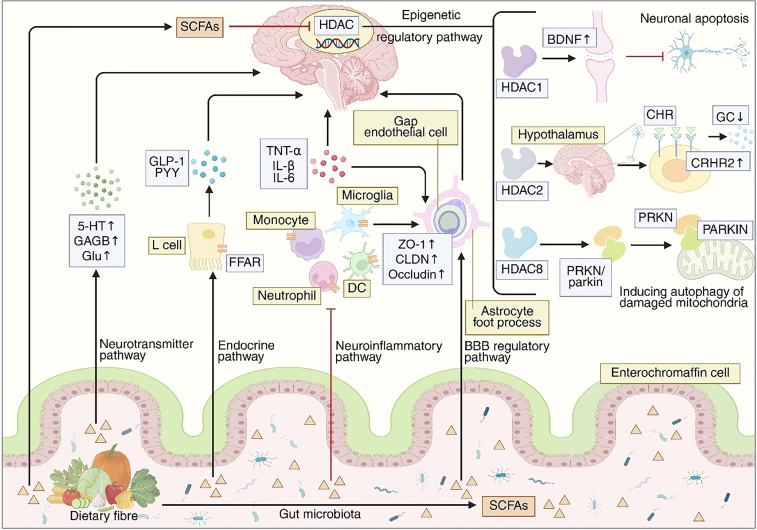
Short-chain fatty acids participate in the gut-brain axis influencing brain physiology. SCFAs produced by probiotic fermentation of dietary fibers can influence brain physiology through neurotransmitter pathway, endocrine pathway, neuroinflammatory pathway, modulation of blood-brain barrier permeability, and epigenetic regulation pathway. SCFAs promote the production and transport of 5-HT, also enter the brain and elevate the levels of GABA and glutamate (Glu). In the gut, SCFAs bind to free fatty acid receptors (FFARs) on enteroendocrine cells (L cells), promoting the secretion of glucagon-like peptide-1 (GLP-1), peptide YY (PYY), and leptin into the systemic circulation, which then transmit signals to the brain. After being absorbed into the bloodstream via monocarboxylate transporters in intestinal cells, SCFAs can inhibit the secretion of proinflammatory factors by binding to FFARs on inflammatory cells such as dendritic cells, macrophages, and microglia, thereby suppressing neuroinflammation. SCFAs can protect the blood-brain barrier (BBB) by either increasing the number of tight junctions between vascular endothelial cells in the barrier or suppressing inflammatory responses. SCFAs entering the brain can inhibit histone deacetylases, thereby promoting brain-derived neurotrophic factor (BDNF) synthesis to protect neurons. It can also promote the expression of parkin protein by the PRKN gene, thereby inducing mitophagy of damaged mitochondria or by upregulating the expression of hypothalamic corticotropin-releasing hormone receptor type 2; 5-HT, 5-hydroxytryptamine; CHR, corticotropin-releasing hormone receptor; CLDN, claudin; CRHR2, corticotropin-releasing hormone receptor type 2; DC, dendritic cell; GABA, γ-aminobutyric acid; GC, glucocorticoid; GLP-1, glucagon-like peptide-1; Glu, glutamate; HDAC, histone deacetylase; PYY, peptide YY; SCFA, short-chain fatty acid; TNF-α, tumor necrosis factor-alpha; ZO-1, zonula occludens-1.

Overall, SCFAs act as pivotal mediators linking gut microbiota metabolism to CNS homeostasis (Fig .1). Through epigenetic regulation, modulation of neuroinflammation, and influence on neurotransmitter synthesis, these microbial metabolites orchestrate multiple pathways that preserve neuronal integrity and cognitive function. Disruption of SCFA production, whether due to microbial dysbiosis or dietary imbalance, therefore represents a critical mechanism contributing to neurodegenerative progression. Restoring SCFA-mediated gut-brain signaling may thus provide a promising therapeutic avenue for mitigating cognitive decline in NDDs.

### FAs and BBB permeability

BBB, a critical neurovascular unit, is composed of continuous capillary endothelial cells sealed by tight junctions, encased by an intact basement membrane, and structurally supported by the end feet of astrocytes. This highly selective barrier system maintains a stable microenvironment essential for proper CNS function (65). Recent studies have shown that BBB permeability is dynamically regulated by metabolic byproducts, among which FAs play a pivotal role in preserving BBB integrity by modulating tight junction protein expression and neuroinflammatory responses (66) (Fig .1).

In the BBB, tight junctions between capillary endothelial cells are composed of various proteins, among which claudin-5 (CLDN-5) is the predominant component. Other key proteins include CLDN-12, occludin, junctional adhesion molecules, and zonula occludens proteins (ZO-1 and ZO-2) (67). Different types of FAs exert distinct regulatory effects on the expression of tight junction proteins. For example, PA—the most abundant saturated LCFA in the human body—can significantly downregulate the expression of ZO-1 and occludin by activating the lead-induced JNK pathway (68). This disruption of BBB integrity impairs the clearance of pathological protein aggregates such as Aβ and α-synuclein (α-Syn) (69, 70, 71), and facilitates the infiltration of endogenous blood components (e.g., fibrinogen, thrombin, and complement proteins) into the brain parenchyma (72), thereby accelerating the progression of NDDs. In contrast, SCFAs produced by microbial fermentation exert protective effects on BBB tight junctions. Braniste *et al*. found that germ-free mice showed markedly reduced occludin and CLDN-5 expression compared to conventionally colonized mice, independent of vascular density or pericyte coverage. Oral sodium butyrate supplementation or colonization with *Clostridium butyricum* restored this expression, directly demonstrating that butyrate strengthens tight junctions and reduces BBB permeability (73). Similarly, propionate indirectly restores ZO-1 expression through free fatty acid receptor (FFAR)2-mediated histone acetylation to modulate T cells (74). Furthermore, in AD model mice, n-3 PUFAs have been demonstrated to effectively reverse the downregulation of ZO-1 and reduce abnormal Aβ deposition in the brain (75). Although the precise mechanisms by which butyrate and n-3 PUFAs regulate tight junction proteins remain to be fully elucidated, possible pathways include activation of GPR109A within the BBB (66) and HDAC inhibition (66, 73).

Notably, increased BBB permeability is closely linked to neuroinflammation, suggesting that FAs may upregulate tight junction proteins partly via anti-inflammatory effects. SCFAs and n-3 PUFAs further enhance BBB integrity by modulating neuroinflammation. For instance, studies have demonstrated that n-3 PUFAs alleviate IL-1β–induced neuroinflammation in a human BBB model by suppressing the NF-κB signaling pathway, thereby improving postoperative cognitive dysfunction in mice (75, 76). Moreover, Hoyles *et al*. revealed that propionate can directly act on FFAR3 expressed on brain endothelial cells, suppressing toll-like receptor 4 (TLR4)-specific signaling and significantly downregulating CD14 mRNA expression, thus protecting the BBB from inflammatory damage (77).

### FAs and lipid peroxidation

As key components of membrane PLs, PUFAs play a critical role in maintaining membrane fluidity and facilitating signal transduction. However, their molecular structure—characterized by multiple unsaturated double bonds—renders them highly susceptible to ROS attack. Under oxidative stress, this vulnerability triggers a cascade of LPO, generating reactive lipid metabolites that compromise membrane integrity, disrupt ionic homeostasis, and covalently modify biomacromolecules. These changes can lead to abnormal protein aggregation and dysregulated signaling pathways. Characteristic markers of LPO have been consistently detected in biological samples (including plasma, cerebrospinal fluid, and brain tissue) from patients with NDDs, providing direct evidence for the pivotal role of LPO in neurodegenerative pathology.

Studies have revealed characteristic alterations in the PUFA profile within the brains of patients with PD, notably an enrichment of highly UFAs such as 22:5n-6 and 22:6n-3, accompanied by increased susceptibility to LPO (78). These changes may be driven by ROS generated in response to oligomeric α-Syn aggregation (79). Interestingly, certain conformations of α-Syn may also facilitate the clearance of LPO products (80), suggesting a complex and bidirectional interaction between α-Syn and LPO. In contrast, brain regions with Aβ plaques in AD patients exhibit a reduction in UFAs and a shortened average FA chain length, likely reflecting localized degradation induced by intensified LPO (81). Furthermore, plasma levels of neuroprostanes—biomarkers of LPO—correlate strongly with medial temporal lobe atrophy in early-stage AD (82), while elevated isoprostane concentrations in cerebrospinal fluid are closely associated with tau pathology (83). In ALS, a systemic oxidative stress state is reflected by reduced plasma PUFA levels (84), with pronounced accumulation of the DHA peroxidation product carboxyethylpyrrole observed in the CNS. Genetic studies have further demonstrated that in ALS patients carrying superoxide dismutase 1 (SOD1) mutations, this process is exacerbated by inflammatory cell activation and myeloperoxidase-mediated oxidative injury (85), underscoring the synergistic pathogenic interplay between neuroinflammation and oxidative stress.

In addition to serving as substrates for oxidative damage, FAs can also exert neuroprotective effects by modulating cellular redox homeostasis. Deuterated PUFAs, characterized by their reduced susceptibility to oxidation, have been shown to effectively attenuate brain LPO. n-3 PUFAs enhance cellular antioxidant defenses primarily through activation of the Nrf2 signaling pathway. For instance, ALA has been reported to upregulate the Nrf2/HO-1 axis, thereby reducing the accumulation of ROS and inducible nitric oxide (NO) synthase in the brains of cadmium-exposed mice, while concurrently modulating apoptosis-related pathways to protect neurons (86). At the molecular level, distinct PUFA derivatives exhibit unique antioxidative profiles. The phosphatidylcholine forms of DHA and EPA (DHA-PC/EPA-PC) significantly restore antioxidant markers such as SOD, total antioxidant capacity, GSH, and glutathione peroxidas, while reducing levels of LPO products, NO, and NO synthase, thereby mitigating oxidative stress (87). Interestingly, increased DHA synthesis observed in the neurons of ALS patients may reflect a compensatory antioxidant response (88). While lowering the brain n-6/n-3 PUFA ratio is generally considered protective (89), certain n-6 PUFAs, such as conjugated LA, also demonstrate antioxidative potential (90), potentially via mechanisms involving NADPH oxidase regulation (91). Moreover, SCFAs such as valerate and propionate have also been reported to reduce ROS levels and oxidative stress (92), further supporting the antioxidative role of microbial metabolites.

### FAs and ferroptosis

**Fig. 2:**
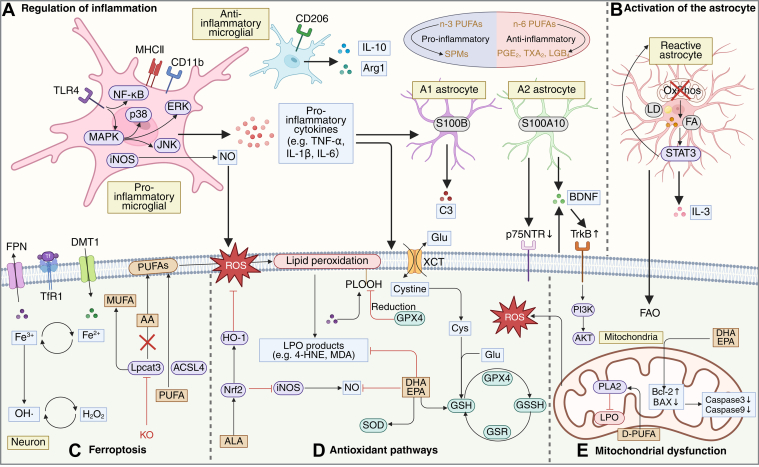
Mechanistic network of fatty acid-mediated regulation of oxidative stress and neuroinflammation. A: Regulation of inflammation. n-3 PUFAs and their specialized proresolving mediators (SPMs) exhibit anti-inflammatory effects by promoting the polarization of microglia from proinflammatory phenotype to anti-inflammatory phenotype. This involves inhibition of TLR4 signaling, downregulation of NF-κB, MAPK, iNOS, and reduction of NO and proinflammatory cytokines (e.g., TNF-α, IL-1β, IL-6), while increasing IL-10 and Arg1 expression. In contrast, n-6 PUFA metabolites (PGE_2_, TXA_2_, LTB_4_) exhibit proinflammatory effects. M1 microglia-derived cytokines further promote the formation of A1 reactive astrocytes, illustrating cross-talk between glial cells. B: Activation of the astrocyte. Impaired mitochondrial oxidative phosphorylation (OxPhos) in astrocytes leads to lipid droplet (LD) accumulation and FA buildup, promoting STAT3 acetylation and reactive astrocyte formation. These astrocytes exhibit suppressed oxidative phosphorylation and iNOS expression and secrete IL-3, which activates microglia and contributes to the neuroinflammatory cycle. In contrast, A2 astrocytes release BDNF and enhance TrkB signaling, supporting neuronal survival via the PI3K/AKT pathway. C: Ferroptosis. Iron uptake via transferrin receptor 1 (TfR1) and divalent metal transporter 1 (DMT1), and export via ferroportin (FPN), regulate intracellular iron levels. Fe^2+^ catalyzes the Fenton reaction to produce hydroxyl radicals (•OH), driving lipid peroxidation. Under the activity of ACSL4 and LPCAT3, PUFAs are incorporated into membranes and oxidized into phospholipid hydroperoxides (PLOOH), while MUFAs inhibit this process. Knockout of LPCAT3 reduces arachidonic acid (AA) incorporation and increases MUFA levels, protecting against ferroptosis. D: Antioxidant pathways. Cystine imported via the XCT antiporter is converted to cysteine and used for GSH synthesis. GSH is utilized by GPX4 to detoxify PLOOH and suppress lipid peroxidation. n-3 PUFAs such as DHA and EPA activate the Nrf2/HO-1 pathway, inhibit iNOS and NO production, reduce oxidative damage, and restore antioxidant enzyme activities including GPX4 and SOD, maintaining redox balance. E: Mitochondrial dysfunction. Mitochondria are major sources of ROS. Lipid peroxidation (LPO) of mitochondrial membranes exacerbates ROS production in a feedback loop. D-PUFAs protect against LPO and mitochondrial dysfunction. DHA and EPA reduce mitochondrial apoptosis by modulating Bcl-2/BAX ratios and suppressing caspase-3/9 activation. 4-HNE, 4-hydroxynonenal; ACSL4, acyl-CoA synthetase long-chain family member 4; ALA, alpha-linolenic acid; BDNF, brain-derived neurotrophic factor; Cys, cysteine; DMT1, divalent metal transporter 1; D-PUFA, deuterated polyunsaturated fatty acid; FAO, fatty acid oxidation; FPN, ferroportin; GLU, glucose; GPX4, glutathione peroxidase 4; GSH, glutathione; GSR, glutathione reductase; GSSG, glutathione disulfide; iNOS, inducible nitric oxide synthase; KO, knockout; LD, lipid droplet; LPCAT3, lysophosphatidylcholine acyltransferase 3; LPO, lipid peroxidation; LTB4, leukotriene B4; MDA, malondialdehyde; MHCⅡ, major histocompatibility complex class II; MUFA, monounsaturated fatty acid; n-3 PUFAs, omega-3 polyunsaturated fatty acids; NO, nitric oxide; OxPhos, oxidative phosphorylation; PGE2, prostaglandin E2; PLA2, phospholipase A2; ROS, reactive oxygen species; SOD, superoxide dismutase; SPMs, specialized proresolving mediators; TLR4, toll-like receptor 4; TNF-α, tumor necrosis factor-alpha; TrF1, transferrin receptor 1; TXA2, thromboxane A2.

Lack of reducing equivalents such as NADPH and GSH may create a unique pathological microenvironment characterized by heightened susceptibility to ferroptosis (93). This recently identified form of regulated cell death is driven by iron-catalyzed peroxidation of PUFA-containing PLs, initiated when ROS react with the labile iron pool via the Fenton reaction. Ferroptosis is usually marked by the accumulation of PL hydroperoxides and depletion of the system Xc^-^/GSH/GPX4 axis (94, 95) (Fig .2).

In the brains of AD patients, the expression of acyl-CoA synthetase long-chain family member 4—a key enzyme in PUFA metabolism—is significantly upregulated, while genes involved in iron homeostasis, such as ferritin, are downregulated (96). In ALS, plasma levels of ferroptosis biomarkers such as 4-hydroxynonenal are elevated and positively correlate with disease progression (97). Sarparast *et al*. (98) found that metabolic products of n-6 FA dihomo-γ-linolenic acid can significantly induce ferroptosis-mediated neurodegeneration. Moreover, Angelova *et al*. demonstrated that in human induced pluripotent stem cell (iPSC-derived neurons with SNCA triplication, targeted inhibition of LPO mitigates α-Syn–induced membrane damage and ROS generation, thereby reducing ferroptotic cell death and highlighting the initiating role of LPO in this process (99). Importantly, ferroptosis involves multiple enzymatic players. In a PD mouse model treated with MPTP, increased expression of acyl-CoA synthetase long-chain family member 4 in the substantia nigra was observed, and its inhibition improved both neuropathology and behavioral deficits—likely through suppression of lipid ROS levels (100). Another study revealed that knockout of lysophosphatidylcholine acyltransferase 3, a key enzyme in PL remodeling, prevented AA incorporation into membrane PLs in a mouse model at the stage of extensive Aβ plaque deposition. This was accompanied by increased de novo lipogenesis of MUFA, reduced levels of LPO byproducts such as 4-hydroxynonenal and acrolein, enhanced microglial clearance of Aβ plaques, and attenuated induction of antioxidant enzymes (101).

### FAs and mitochondrial dysfunction

As the central hub of cellular energy metabolism, mitochondria are particularly vulnerable in NDDs, and the dysfunction of which may in turn exacerbate the pathological progression (Fig .2). For example, LPO disrupts mitochondrial membrane integrity and impairs the electron transport chain, initiating a vicious cycle of impaired ATP synthesis and excessive ROS production (102). Proteomic analyses of PD brains reveal that progressive mitochondrial dysfunction precedes α-Syn aggregation, characterized by respiratory chain defects, acyl-CoA acetyltransferase 1 cholesterol acyltransferase 1-mediated enhancement of FAO, and redox imbalance (103). Additionally, mitochondria in astrocytes—key sites for FAO—are susceptible to oxidative phosphorylation deficits, leading to impaired FAO, LD accumulation, and STAT3 acetylation, which drive reactive astrogliosis. This, in turn, can induce compensatory activation of FAO and oxidative stress responses in neurons, further promoting AD pathology (104). Interestingly, stress granules formed during starvation interact with mitochondria and LDs, suppressing FAO by downregulating voltage-dependent anion channel proteins, which promotes FA storage in LDs and reduces oxidative stress (105).

Notably, certain FAs have shown the ability to affect mitochondrial membrane integrity, as well as regulate mitochondrial dynamics and mitochondria-dependent apoptosis, thus reverse the pathological changes and restore mitochondrial function. For instance, propionate supplementation in AD model mice can improve cognitive function by modulating G protein–coupled receptors. It reduces Drp1 expression and promotes mitochondrial fission in hippocampal neurons via FFAR3-dependent inhibition of ERK phosphorylation, while also enhancing PINK1/PARKIN-mediated mitophagy through FFAR2 activation (9). Prophylactic treatment with valproic acid in cultured cells protects against Aβ25-35–induced cytotoxicity by increasing the Bcl-2/BAX ratio, suppressing ROS production, and enhancing SOD activity. It further prevents mitochondrial membrane potential loss, inhibits cytochrome c release, promotes ATP synthesis, and significantly improves mitochondrial dysfunction (106). Similarly, DHA- and EPA-conjugated phosphatidylserine (DHA-PS/EPA-PS) can alleviate oxidative damage induced by H_2_O_2_/t-BHP in primary hippocampal neurons by regulating Bcl-2/BAX and its downstream caspase family, affecting the TrkB/ERK/CREB signaling pathway (107). Meanwhile, increasing endogenous n-3 PUFAs or coincubating cells with DHA alleviates Aβ-induced mitochondrial fragmentation and restores mitochondrial respiration (108), potentially through upregulation of TET activity and 5-hydroxymethylcytosine levels (109). Similarly, treatment with butyrate in ALS cellular models has been shown to improve mitochondrial function, possibly by activating the PGC1α signaling axis (110).

### FAs and neuroinflammation

Neuroinflammation is recognized as a hallmark of NDDs, closely linked to disease onset and progression. Growing evidence indicates that FAs and their lipid-derived mediators—serving as both metabolic substrates and signaling regulators—play significant roles in the modulation of neuroinflammation (111).

Among PUFAs, AA, an n-6 PUFA, gives rise to proinflammatory eicosanoids such as prostaglandins, leukotrienes, and thromboxanes. These metabolites activate microglia and promote the release of inflammatory cytokines, thereby exacerbating neuronal injury (112, 113). In contrast, n-3 PUFAs are primarily metabolized into specialized proresolving mediators (SPMs)—including resolvins, protectins, and maresins—that exert anti-inflammatory and neuroprotective effects (114). Meanwhile, certain n-3 PUFAs can also compete with AA for the same metabolic enzymes, indirectly suppressing the production of proinflammatory n-6 eicosanoids (115). Under pathological conditions, the metabolic balance between PUFAs and SPMs is disrupted. For instance, a reduced n-3/n-6 PUFA ratio, along with decreased levels of the DHA-derived neuroprotectin D1 and activated eicosanoid signaling, is observed in the brains of AD patients—particularly in APOE4 carriers—suggesting a state of chronic inflammation. Further lipidomic analysis suggests that these changes are associated with activation of calcium-dependent phospholipase A2, 5-lipoxygenase, and soluble epoxide hydrolase (116).

Beyond their metabolites, FAs themselves can regulate neuroinflammation by acting on glial cells and indirectly influencing neuronal survival and integrity. The protective role of n-3 PUFAs is increasingly well established. Dietary supplementation with DHA- and EPA-rich fish oil in fat-1 transgenic mice reduces the expression of inflammation-related genes induced by Aβ, independent of changes in lipid mediators (117). In an ALS mouse model, DHA supplementation elevates DHA and reduces AA levels in the lumbar spinal cord, increases the anti-inflammatory index, and lowers plasma TNF-α concentrations (118). n-3 PUFAs such as EPA, DHA, and ALA promote microglia to switch from anti-inflammatory phenotype to proinflammatory phenotype (89, 119, 120, 121, 122). Similarly, in astrocytes, n-3 PUFAs downregulate A1 neurotoxic markers while restoring A2 neurotrophic phenotypes (123). This shift supports neuronal survival and synaptic function through activation of the brain-derived neurotrophic factor (BDNF)/TrkB-PI3K/AKT pathway (89, 121), accompanied by increased expression of the high-affinity BDNF receptor TrkB and decreased expression of the low-affinity receptor p75NTR (86, 124). Notably, across multiple models, n-3 PUFA administration reduces Iba-1 and GFAP expression—markers of activated microglia and astrocytes, respectively (121, 122, 124)—suggesting both normalization of glial polarization and attenuation of glial proliferation under neuroinflammatory conditions (125). In addition, Wen *et al*. showed that EPA-PC supplementation in AD rats suppresses Aβ-induced neurotoxicity by attenuating NLRP3 inflammasome activation (126), underscoring the involvement of the innate immune system in neuroinflammation.

Conversely, certain FAs can directly trigger neuroinflammatory responses. HFD, rich in SFAs, increases hippocampal TNF-α and IL-1β levels in mice. Treatment of neuronal (N2A) and microglial (BV2) cell lines with PA, a major HFD component, induces gene-level inflammatory changes within 10 days (127). PA has been shown to activate NF-κB signaling and c-Jun phosphorylation in microglia, upregulate TNF-α, IL-6, and inducible NO synthase expression, and exert paracrine cytotoxic effects on neurons. These adverse effects are mitigated by the MUFA OA (C18:1 n-9) (128). Moreover, in astrocytes with impaired FA degradation, excessive FA accumulation increases acetyl-CoA levels, activating STAT3 and driving reactive astrocyte formation. These reactive astrocytes can then activate microglia via IL-3 signaling, further propagating neuroinflammation (104).

In conclusion, FAs are key regulators of neuroinflammation in NDDs, with distinct effects depending on their types and metabolic derivatives (Fig. 2). Elucidating the molecular mechanisms and signaling pathways by which FAs mediate neuroinflammatory responses may yield novel therapeutic targets and strategies for the prevention and treatment of NDDs.

## Disease-specific roles of FAs in NDDs

Building on the shared regulatory mechanisms of FAs in NDDs, emerging evidence indicates that distinct FA subtypes exert disease-specific effects. In major NDDs such as AD, PD, and ALS, n-3/n-6 PUFAs, SCFAs, and MCFAs participate in key pathological processes through unique pathways—including Aβ deposition and tau phosphorylation in AD, α-Syn aggregation and mitochondrial dysfunction in PD, and neuromuscular junction (NMJ) degeneration and glutamate excitotoxicity in ALS. These observations underscore the disease-specific regulation of FA metabolism. This section delineates the distinct mechanistic roles of these FA subtypes in AD, PD, and ALS, providing a theoretical basis for targeted FA–based therapeutic strategies.

### FAs and AD

As the most prevalent NDD, AD is characterized by a multifactorial pathogenesis involving Aβ deposition, tau hyperphosphorylation, neuroinflammation, and oxidative stress (129). In the critical pathway of Aβ production, the β-site amyloid precursor protein (APP) lyase 1 (BACE1) is responsible for cleavage of APP, producing a soluble APP extracellular domain and a membrane-bound C99 fragment, which is then cleaved into Aβ protein by γ-secretases (composed of prosenin 1 or prosenin 2) on the cell membrane (130). The activity and expression level of BACE1 are key rate-limiting steps in regulating the rate of Aβ production. In addition, APP can also be hydrolyzed by α-secretases to ultimately prevent the production of Aβ (131). Recent studies have identified dysregulated FA metabolism as a critical contributor to AD progression. Notably, significant alterations in the levels of PUFAs, MUFAs, and SCFAs have been observed. These changes influence disease pathology through multiple mechanisms, including modulation of Aβ accumulation and antioxidant capacity (3).

In the course of AD, Aβ deposition is considered to be one of the core mechanisms of the disease, so targeted regulation of Aβ is considered a potential therapeutic strategy for AD. Studies have shown that DHA and OA have the role of regulating Aβ production–related pathways. In AD animal models, DHA has been found to attenuate hippocampal Aβ deposition by downregulating the expression of APP, presenilin 1 (PS1), and BACE1 (87). In addition, OA plays a key role in inhibiting Aβ deposition (38). OA promotes non-amyloid production and processing of APP through α-secretase and significantly inhibits the activity of BACE 1, thereby reducing Aβ production (39).

It is worth noting that the neuroprotective potential of DHA shows significant individual differences in clinical practice. In individuals with mild cognitive impairment (MCI) who carry the APOE ϵ4 allele, early DHA supplementation can delay the progression to AD, with its effectiveness positively correlated with cerebrospinal fluid DHA levels. Conversely, in later stages of AD, DHA supplementation tends to yield greater benefits in APOE ϵ4 noncarriers (132).

Notably, the effects of SCFAs in AD models appear to be complex and biphasic. Colombo *et al*. (133) reported that in germ-free APP/PS1 mice, SCFA supplementation unexpectedly exacerbated Aβ deposition and was associated with increased APOE expression in microglia. This finding suggests that the neuroprotective effects of SCFAs are highly dependent on dosage and pathological context, with mechanisms that remain to be fully elucidated. Collectively, these insights underscore the complexity of FA metabolism in AD therapy. Future research should account for FA subtype, dosage, and disease stage to guide the development of more precise and effective therapeutic strategies.

### FAs and PD

PD is a neurodegenerative disorder characterized by the degeneration of dopaminergic neurons in the substantia nigra-striatal pathway, with core pathological features involving abnormal aggregation of α-Syn and dopaminergic neuronal damage (134). α-Syn is a protein of 140 amino acids encoded by SNCA, which is mostly found in the presynaptic membrane and plays a role in regulating neurotransmitter release (135). Under physiological conditions, soluble α-Syn can be degraded by the autophagy pathway (136). Environmental factors such as SNCA point mutations or oxidative stress can cause α-Syn to misfold and aggregate with each other to form oligomers, which in turn can form Lewy bodies and produce cytotoxic damage to dopamine neurons (135). Increasing evidence indicates that alterations in SCFAs and PUFAs are closely associated with α-Syn aggregation (137).

Sodium butyrate supplementation has been shown to enhance the autophagic degradation of α-Syn by upregulating the expression of the autophagy-related protein Atg5 (138), while propionate inhibits downstream α-Syn signaling pathways (139, 140). DHA can reduce the aggregation of α-Syn in the enteric nervous system (141).

In addition, sodium butyrate can also protect dopaminergic neurons by inhibiting neuroinflammation in PD patients (138). Importantly, the effects of propionate extend beyond the CNS—it can activate FFAR3 in the enteric nervous system and mediate the neuroprotective actions of osteocalcin, thereby mitigating dopaminergic neuron loss in PD mice (142).

However, the effects of SCFAs in PD are not universally beneficial. Sampson *et al*. reported that in germ-free α-Syn–overexpressing mice, SCFA supplementation exacerbated neurodegeneration and motor deficits by activating microglia and promoting α-Syn aggregation (6). The increased inflammation observed in germ-free α-Syn–overexpressing mice may result from heightened microglial sensitivity under germ-free conditions combined with locally elevated SCFA concentrations (7).

### FAs and ALS

ALS is a rare and fatal neurodegenerative disorder primarily characterized by the progressive degeneration of motor neurons. Although its precise pathogenesis remains incompletely understood, NMJ degeneration, disrupted energy metabolism, and oxidative stress are all closely implicated. In recent years, the regulatory role of FA metabolism in ALS progression has attracted growing research attention (143).

One of the core features of ALS pathology is metabolic dysfunction, particularly a hypermetabolic state in skeletal muscle and a metabolic shift from glucose utilization to FA βOX (144, 145). Notably, this shift can be detected even before the onset of clinical symptoms. Sapaly and colleagues found that dysfunction of mitochondrial cholesterol transport proteins NPC1 and NPC2 is closely associated with energy imbalance in ALS (146). Concurrently, excessive activation of FA βOX contributes to mitochondrial overproduction of ROS, thereby aggravating neuronal oxidative damage (147). As the disease progresses, mitochondrial dysfunction caused by impaired metabolism of carnitine and short-chain acylcarnitine is gradually inhibited (145).

NMJ degeneration is recognized as an early pathological hallmark of ALS and may precede clinical manifestation (148). NMJ degeneration includes presynaptic injury, disruption of synaptic cleft and postsynaptic injury, among which presynaptic injury is mainly neurotransmitter release disorder, and postsynaptic injury is mainly transmitter receptor cluster disintegration (149). MCFAs, such as nonanoic acid and 4-methyloctanoic acid, significantly ameliorate motor neuron and NMJ defects in ALS fly models. Mechanistic studies suggest that nonanoic acid protects NMJs by modulating the morphology of postsynaptic type IIA glutamate receptors, while 4-methyloctanoic acid improves synaptic function by enhancing presynaptic neurotransmitter release (150). Additionally, caprylic acid has been shown to alleviate motor deficits in ALS models by promoting motor neuron excitability (151).

These studies highlight the highly heterogeneous regulatory roles of FAs across different NDDs. In AD, DHA mitigates cognitive decline through dual mechanisms involving the inhibition of Aβ deposition and antioxidant enhancement. In PD, SCFAs support dopaminergic neuron function by activating autophagy and modulating the gut-brain axis. In ALS, MCFAs exert neuroprotective effects by restoring NMJs and reshaping energy metabolism. Despite these disease-specific regulatory targets, FAs also exhibit cross-disease actions by modulating shared pathological pathways (Table 1).

**Table 1:** Latest advances in the role of fatty acids in the core mechanisms of NDDs

Disease	Core Mechanism	Fatty Acid Types	Conclusion	Citation
AD	Characteristic pathological features	n-3 PUFA (DHA)	DHA was significantly lower in MCI relative to control subjects in plasma/serum.	(3)
		SCFA (BA)	Dietary SCFA reduce amyloid deposition and tau protein hyperphosphorylation in elderly APP/PS1 mice. -SCFA-contained ACM was able to restore neuronal glutaminase abnormal expression	(64)
	Gut-brain axis	SCFA (BA)	Disturbance of short-chain fatty acid metabolism led to high HDAC4 expression in the hippocampus to promote increased neuronal apoptosis.	(48)
		SCFA (an equimolar mixture of Na-acetate, Na-propionate, and Na-butyrate)	Germ-free (GF) AD mice exhibit a substantially reduced Aβ plaque load and markedly reduced SCFA plasma concentrations	(133)
		n-3 PUFA (EPA)	Short-term EPA supplementation shaped the gut microbiota by increasing butyrate-producing bacteria of the Firmicutes phylum It also downregulated the inflammatory microglial marker MHCII in two distinct regions of CNS.	(120)
	BBB permeability	SCFA	SCFAs treatment can improve the integrity of the blood-CSF barrier in AD mice and this is accompanied by a pronounced reduction in Aβ load	(179)
		n-3 PUFA (DHA)	Treatment of fish oil for 4 weeks remarkably increased ZO-1 expression and significantly inhibited the glial activation.	(75)
	Oxidative damage	n-6 PUFA (CLA)	CLA pretreatment is likely to downregulates/prevents the noxious effects of Al through the activation of an Nrf2-mediated adaptive response. CLA protects against H-induced decline of mitochondrial complex activity and increased protein oxidation.	(90)
		n-6 PUFA (CLA)	The mixture (50:50) of two geometric and positional isomers of linoleic acid prevents AlCl3-induced UPR, Nrf2 hyperactivation, and maladaptive response.	(91)
		n-6 PUFA (AA)	The reduction of AA in microglia significantly ameliorated oxidative stress and inflammatory responses while enhancing the phagocytosis of Aβ plaques and promoting the compaction of Aβ deposits.	(101)
	Neuroinflammation	SCFA (Ac)	Acetate is able to modulate microglial phagocytosis and disease progression during neurodegeneration.	(49)
		SCFA (Ac)	Acetate inhibited the phosphorylation of NF-κB p65, ERK, and JNK and decreased the levels of COX-2 and interleukin 1β in the Aβ-stimulated BV2 microglia.	(50)
		FA	Intercellularly, lipid-laden reactive astrocytes stimulate neuronal FA oxidation and oxidative stress, which activate microglia via IL-3 signaling.	(104)
		n-3 PUFA (ALA)	In the Aβ + ALA-cotreated group, TLR4, GFAP, Iba-1, p-JNK, p-NF-kB p65 (Ser536), and TNF were significantly reduced.	(122)
PD	Characteristic pathological features	SCFA (BA)	NaB causes α-Syn degradation by an Atg5-dependent and PI3K/Akt/mTOR-related autophagy pathway.	(138)
		SCFA (PA)	Propionate supplementation suppresses neurodegeneration without reducing α-synuclein aggregation.	(139)
		n-3 PUFA (EPA)	EPA is able to reduce the neurotoxic effect of neurotoxin 6-hydroxydopamine (6-OHDA) in vitro and increase the expression of BDNF and GDNF by decreasing methylation levels.	(180)
	Gut-brain axis	SCFA (PA)	OCN ameliorates motor deficits and dopaminergic neuronal loss in PD mice	(142)
	Oxidative damage	n-3 PUFA (DHA)	DHA can increase Nrf2 levels and decreases n-6 PUFA-derived metabolites to reduce oxidative stress.	(181)
		PUFA	α-Syn oligomers can induce lipid peroxidation.	(99)
		PUFA	Knockdown of ACSL4 in the SN specifically prevented the lipid ROS elevation without affecting the mitochondrial ROS changes.	(100)
	Neuroinflammation	SCFA (BA)	B. product-derived butyrate causally inhibited activation of microglia of PD by regulating RAS-NF-κB pathway.	(182)
		SCFA	SCFAs were found to exacerbate motor and gastrointestinal dysfunctions in PD models, intensifying α-Syn pathology and neuroinflammation.	(183)
ALS	Characteristic pathological features	MCFA (NA 4-MOA)	NA modified postsynaptic glutamate receptor density, whereas 4-MOA restored defects in the presynaptic vesicular release.	(150)
	Oxidative damage	FA	SG formation leads to a downregulation of FAO through the modulation of mitochondrial VDACs, which import FAs into mitochondria, reducing oxidative damage.	(105)
		SCFA (BA)	NSC34-G93A cells treated with BA showed an increase in mitochondrial spare respiratory capacity with elevated maximal respiration. The time-dependent changes in the mRNA level of PGC1α revealed a burst induction. Both the mRNA and protein levels of the key molecules (MTCO1, MTCO2, and COX4) related to the mitochondrial electron transport chain were increased.	(110)
	Neuroinflammation	n-3 PUFA (DHA)	DHA supplementation led to an increased anti-inflammatory fatty acid profile (ca 30%, *P* < 0.01) and a lower concentration of circulating proinflammatory cytokine TNF-α (*P* < 0.001 in males).	(118)

4-MOA, 4-methyloctanoic acid; 6-OHDA, 6-hydroxydopamine; AA, arachidonic acid; Ac, acetate; MCI, mild cognitive impairment; ACM, astrocyte-conditioned medium; ACSL4, acyl-CoA synthetase long-chain family member 4; AD, Alzheimer's disease; ALA, α-linolenic acid; ALS, amyotrophic lateral sclerosis; APP, amyloid precursor protein; Aβ, β-amyloid; α-Syn, α-synuclein; BA, butyric acid; BDNF, brain-derived neurotrophic factor; CLA, conjugated linoleic acid; CNS, central nervous system; COX-2, cyclooxygenase-2; FAO, fatty acid oxidation; FFAR3, free fatty acid receptor 3; GDNF, glial cell line–derived neurotrophic factor; GF, germ-free; HDACs, histone deacetylases; LPLs, lipoprotein lipases; MCFA, medium-chain fatty acid; n-3 PUFA, omega-3 polyunsaturated fatty acid; OA, oleic acid; OCN, osteocalcin; OxPhos, oxidative phosphorylation; PD, Parkinson's disease; PS1, presenilin 1; ROS, reactive oxygen species; SCFA, short-chain fatty acid; SG, stress granules; SN, substantia nigra; SPF, specific pathogen-free; TLR4, toll-Like receptor 4; TNF-α, tumor necrosis factor-α; UPR, unfolded protein response; VDAC, voltage-dependent anion channel.

## Future research directions for FA-based therapies in NDDs

### Targeted modulation of the microbiota-gut-brain axis

In strategies targeting the microbiota-gut-brain axis (MGBA), probiotics, prebiotics, and fecal microbiota transplantation (FMT) are the most actively explored measures in current research. FMT has been reported to significantly affect neurological inflammation, intestinal barrier integrity, and behavioral outcomes by modulating various levels of the MGBA.

In PD mouse models, FMT reverses gut microbiota imbalance caused by rotenone and improves GI and motor function. Zhao *et al*. (152) found that FMT markedly suppresses the TLR4/MyD88/NF-κB signaling pathway in the gut and substantia nigra, thereby reducing systemic inflammation and dopaminergic neuronal damage to exert neuroprotective effects. Mechanistic studies further revealed that FMT reduces LPS concentrations in both the intestine and serum, suppressing activation of the TLR4/MyD88/NF-κB pathway in the gut and substantia nigra, thereby mitigating inflammation and preserving dopaminergic neurons. In addition, transplantation of donor microbiota enriched with *Lactobacillus murinus* in PD mice has been shown to replicate, at least in part, the neuroprotective effect of fucoidan, indicating that this strain might be pivotal in modulating inflammation and behavioral phenotypes (153).

Neuroprotective effects of FMT have also been observed in AD models. A multiomics study revealed that FMT regulates host glycerophospholipid metabolism, alleviates astrocyte and microglial activation, and improves cognitive performance in APP/PS1 transgenic mice (154). A metagenomic analysis across different AD stages indicated that FMT might be effective during preclinical phases, particularly prior to mild cognitive impairment, by altering microbial metabolism and influencing neuroinflammatory responses (155). Moreover, the gut microbiota is closely associated with the reactivity of glial cells. Under an Aβ pathological condition, microbiota depletion via antibiotics resulted in fewer GFAP^+^ astrocytes with normalized morphology and decreased C3 expression in APP/PS1 mice; FMT partially restored these parameters, suggesting that gut microbiota regulates astrocyte activation via both microglial-dependent and independent pathways (156).

Although current research generally supports the beneficial effects of FMT, some studies have reported potential adverse outcomes. In rotenone-induced PD mice, FMT increases CXCL1 expression in the serum and substantia nigra, leading to early neuroinflammation and iron deposition, suggesting that microbiota from specific sources may exacerbate neuropathology under certain conditions (157). A separate investigation into the inverse association between AD and colorectal cancer revealed that AD-related microbiota possess anticarcinogenic properties, yet the induced “inflammatory tolerance” state was linked to cognitive deterioration, underscoring the context-dependent and tissue-specific effects of gut microbes (153).

In summary, the action of FMT in neurodegenerative disorders mainly relies on its ability to regulate neuroinflammation, metabolic pathways, and immune responses via the gut microbiota. Nevertheless, the effectiveness of FMT varies depending on factors such as donor microbiota characteristics, disease subtype and progression, and the timing of therapeutic delivery. Further research is needed to pinpoint critical microbial taxa and mechanisms, refine donor screening and FMT protocols, and comprehensively assess its long-term safety and therapeutic boundaries to support clinical translation and personalized treatment strategies.

### Dietary and nutritional interventions for FA metabolism regulation

Dietary and nutritional interventions exert a critical role in the prevention and treatment of NDDs by regulating lipid metabolism, inflammation, and neuroprotective mechanisms. Research on essential FA supplementation, specific dietary patterns, and the development of functional FA derivatives is gradually uncovering the complex links between lipid metabolism and NDDs.

Appropriate supplementation of essential FAs, particularly PUFAs, is well known to have neuroprotective effects. In contrast, excess n-6 FAs may exacerbate neuroinflammation and ferroptosis (98). Beyond natural PUFAs, their derivatives have also shown emerging therapeutic promise in NDDs. For instance, n-3 FA derivatives like resolvin D1 and neuroprotectin D1 have shown excellent antioxidant and antiapoptotic effects in multiple models of neurodegeneration, and some have entered early clinical trials (158, 159). FA receptor agonists such as FFAR1 and FFAR4 exhibit anti-inflammatory effects similar to DHA, offering new targets for drug development (160).

It is noteworthy that specific dietary patterns show significant value in the prevention and treatment of NDDs. The Mediterranean diet, which features olive oil, nuts, fresh fruits and vegetables, whole grains, and fish, is high in MUFAs, dietary fiber, and antioxidants, and has been shown to significantly lower the risk of developing AD and PD (161). Variants like the Mediterranean-ketogenic diet improve neuroinflammation and cognition partly via gut microbiota modulation (e.g., increasing Lactobacillus abundance) (162), while the ketogenic diet enhances FA oxidation and ketone production (163) can reduce inflammation-related lipid enzymes in ALS patients (164).

Intermittent fasting and caloric restriction may also confer neuroprotection by activating autophagy, improving mitochondrial function, and reducing inflammation. Overall, integrated strategies—combining dietary optimization, FA supplementation, and functional derivatives—offer promising approaches to maintain neural metabolic homeostasis and modulate inflammatory responses (Table 2).

**Table 2:** Fatty acid-related dietary and nutritional intervention strategies

Intervention Category	Specific Intervention	Main Components/Features	Fatty Acids Involved	Mechanism of Action/Impact Pathway	Citation
Nutritional intervention					
Supplementation of essential fatty acids	DHA supplementation	n-3 PUFA	DHA	Inhibits M1-type microglial polarization while promoting M2-type polarization. Regulates TLR, MAPK, and NF-κB pathways, reduces inflammation, and protects neurons.	(160)
	EPA supplementation (anti-inflammatory n-3 fatty acid)	High doses of EPA (1500–2000 mg/day) or combined EPA+DHA supplementation	EPA	Improves cognition and reduces inflammation-related marker expression through anti-inflammatory mechanisms.	(184)
Excessive n-6 fatty acid intake	Accumulation of γ-linolenic acid metabolites	n-6 polyunsaturated fatty acids (DGLA metabolites)	γ-linolenic acid Metabolites	Facilitates lipid peroxidation, triggers ferroptosis, and aggravates neurodegenerative processes.	(98)
Short-chain fatty acid supplementation	SCFA supplementation	Microbial metabolites: acetate, propionate, and butyrate	SCFAs	SCFAs induce transcriptional remodeling in microglia, increase Aβ deposition, and modulate APOE expression.	(133)
		Prebiotics and high-fiber diet products (propionate, butyrate, β-hydroxybutyrate, etc.)	SCFAs	Modulates α-Syn aggregation, reduces oxidative stress and neuroinflammation, and regulates ROS/RNS levels.	(14)
		15-week low-fiber diet induction model combined with SCFA supplementation intervention	SCFAs	A low-fiber diet reduces SCFA levels, induces gut microbiota dysbiosis, activates microglia, and enhances synaptic engulfment, which SCFA supplementation can reverse.	(189)
Functional derivatives of fatty acids	Resolvin D1, NPD1	Anti-inflammatory lipid mediators derived from n-3 fatty acids	Resolvins, neuroprotectins	Suppress oxidative stress, apoptosis, and neuroinflammation, promoting neuroprotection and repair.	(185, 200)
Gut-brain axis intervention	Oral delivery of a novel small molecule FLZ	Dependent on gut absorption, modulating TLR4/MyD88/NF-κB pathways.	-	Enhances gut barrier, inhibits inflammation, protects substantia nigra dopaminergic neurons, and exerts gut-brain axis functions.	(190)
Targeting receptors related to fatty acids	Dimethyl itaconate (DI)	Itaconate derivative that provides anti-inflammatory and gut barrier-protective effects.	Butyrate and propionate	Repairs gut barrier, increases SCFA production, suppresses neuroinflammation, and reduces synaptic damage.	(186)
	Semaglutide (GLP-1 receptor agonist)	A GLP-1 receptor agonist (GLP-1RA) for type 2 diabetes that has cerebrovascular protective and anti-inflammatory effects.	-	Enhances BBB integrity, microvascular function, and modulates inflammation in microglia and astrocytes.	(187)
	NLY01 (long-acting GLP-1RA analog)	High brain penetrability, prolonged half-life, and suppression of microglial activation.	-	Targets microglial inflammatory pathways and blocks α-synuclein–induced toxic conversion of astrocytes.	(191)
	LM11A-31 (oral)	Acts on synaptic protection and pathways associated with Aβ and tau toxicity.	Primarily not focused on lipid metabolism but can influence lipid raft-related pathways.	Slows down synaptic loss and glial cell activation, improves FDG-PET and CSF inflammation biomarkers.	(192)
	GPR120 and GPR40 agonists	Receptor agonists (fatty acid sensors)	Indirectly regulate n-3 fatty acid signaling.	Inhibit proinflammatory factor secretion, modulate lipid metabolic pathways, and promote neuroprotection.	(188, 193, 194)
Dietary intervention strategies					
Mediterranean diet	Standard Mediterranean diet	Rich in MUFAs (olive oil), dietary fiber, and polyphenols	MUFA	Enhances gut microbiota composition, reduces systemic inflammation, and elevates anti-inflammatory metabolites	(161)
Mediterranean-ketogenic diet (MkD)	MkD pattern	Combines Mediterranean diet characteristics with ketogenic elements, featuring low carbohydrates and high fats.	Enhances fatty acid metabolism and stimulates ketone body production.	Increases the abundance of Lactobacillus, improves gut microbiota composition, enhances short-chain fatty acid metabolism, and protects cognitive function.	(162)
Ketogenic diet (KD)	High-fat, very low–carbohydrate diet	Various fatty acids and ketone body production	Metabolism of saturated and unsaturated fatty acids	Reduces the expression of COX1, COX2, and ALOX5, decreases the production of proinflammatory lipid mediators (such as PGE2, LTB4), and improves neuroinflammation.	(164)
Intermittent fasting	Structured fasting	Enhances fat metabolism and autophagy	-	Improves mitochondrial function, reduces oxidative stress, and triggers neuroprotective autophagy.	(195)
Multidimensional intervention + n-3 (MAPT Study)	Cognitive training + nutrition counseling + physical activity + DHA+EPA supplementation	DHA+EPA	DHA+EPA	Combined intervention demonstrated a protective trend across various dimensions of “intrinsic capacity,” potentially delaying functional decline.	(196)
Anti-inflammatory prebiotic intervention	Anti-inflammatory dietary fiber	Fermentable dietary fiber (RS-type)	SCFA metabolic products	Increases levels of short-chain fatty acids (such as butyrate), reduces gut inflammation marker calprotectin, and modulates gut microbiota.	(197)
Short-term prebiotic fiber intervention	Short-term prebiotic fiber intervention	A combination of fermentable dietary fibers	SCFA metabolic products	Enhances gut microbiota composition, stimulates SCFA production, reduces inflammation, and improves NfL neuronal injury biomarkers.	(198)
Gut microbiota intervention	Oral administration of *F*. *prausnitzii* strains	Fp360 (live bacteria) and Fp14 (pasteurized bacteria)	Butyrate-producing bacteria	Reduces Aβ-induced cognitive deficits, involving oxidative stress relief and enhanced mitochondrial function.	(199)
	FMT	Restores gut microecology and reduces LPS burden.	-	Regulates the LPS-TLR4-MyD88-NF-κB pathway, reduces inflammation, and improves neural damage.	(152)

AD, Alzheimer's disease; ALOX5, arachidonate 5-lipoxygenase; ALS, amyotrophic lateral sclerosis; BBB, blood-brain barrier; COX1, cyclooxygenase 1; COX2, cyclooxygenase 2; CSF, cerebrospinal fluid; DGLA, dihomo-γ-linolenic acid; FDG-PET, fluorodeoxyglucose positron emission tomography; FLZ, fluorizoline; FMT, fecal microbiota transplantation; GLP-1, glucagon-like peptide-1; LPS, lipopolysaccharide; LTB4, leukotriene B4; MARK, microtubule affinity-regulating kinase; MCI, mild cognitive impairment; MkD, mevalonate kinase deficiency; MUFA, monounsaturated fatty acid; MyD88, myeloid differentiation primary response 88; NfL, neurofilament light chain; NF-κB, nuclear factor kappa-light-chain-enhancer of activated B cells; PD, Parkinson's disease; PEG2, prostaglandin E2; RCT, randomized controlled trial; ROS/RNS, reactive oxygen species/reactive nitrogen species; SCFA, short-chain fatty acid; TLR, Toll-like receptor; TLR4, Toll-like receptor 4.

### Delivery strategies and clinical translation of FA therapies

#### Innovative approaches for targeted FA delivery in NDDs

FAs and their derivatives face major clinical challenges due to their physicochemical limitations, including low water solubility, poor bioavailability, oxidative instability, and limited efficiency in crossing the BBB (165, 166). Consequently, the development of new and effective drug delivery systems is crucial for promoting the translation of FA-based therapies from basic research to clinical practice.

To improve the delivery and central efficacy of FA-related interventions—whether derived from FMT, probiotics, or dietary modulation—novel carrier systems are being developed. Nanocarriers such as liposomes, solid lipid nanoparticles, and polymer-based systems have shown the ability to stabilize FA molecules, prolong their circulation time, and enhance CNS bioavailability (167). These technologies may be particularly useful as adjuncts to microbiota-based therapies, facilitating the systemic or localized delivery of microbial metabolites or FA analogs. Notably, biomimetic nanocarriers have demonstrated therapeutic synergy with host immune and metabolic pathways relevant to the MGBA. For instance, Li *et al*. (168) designed a nanoparticle system that activates microglial TREM2 signaling, enhances lipid turnover, suppresses neuroinflammation, and improves cognition in NDD models—mechanisms that parallel those influenced by beneficial gut-derived FAs. Combining engineered lipid nanocarriers with microbial therapies such as FMT may thus amplify metabolic and immunomodulatory effects along the MGBA.

Moreover, gene engineering technologies are emerging as complementary tools to modulate FA metabolism in the host in a microbiota-responsive manner. For example, CRISPR-CasRx systems and viral vectors have been shown to cross the BBB and regulate glial or neuronal function in vivo (169, 170). While not yet directly utilized for FA delivery, these tools offer precise regulation of host lipid enzymes and signaling axes, thus providing a promising platform for developing synergistic microbiota-lipid metabolic therapies. To conclude, advanced delivery technologies centered on nanomedicine and genetic modulation may help bypass existing bottlenecks in lipid signaling therapies, enhancing their pharmacological performance and neural targeting. Coupling these systems with FMT or gut microbiota interventions could unlock new directions in MGBA modulation and accelerate clinical adoption of FA-based neurotherapies.

#### Clinical studies on FA-based therapies for NDDs

Despite the recognized neuroprotective potential of FAs, their clinical translation in NDDs remains limited. Most current interventions are based on nutritional supplementation, and significant challenges persist in formulation stability, delivery efficiency, and therapeutic consistency—particularly for UFAs such as DHA and EPA, which exhibit poor water solubility, low bioavailability, and limited BBB permeability (165).

Several clinical studies have attempted to evaluate the efficacy of PUFAs, particularly in AD. A randomized controlled trial by Tofiq *et al*. demonstrated that daily supplementation with 2.3 g of n-3 FAs (1.7 g DHA and 0.6 g EPA) increased cerebrospinal fluid biomarkers such as YKL-40 and neurofilament light chain, which are associated with neuroinflammation and neuronal injury. However, these biomarker changes were not significantly correlated with cognitive scores (mini-mental state examination), indicating the need for larger, multicenter trials to establish clinical benefits (171). In the context of ALS, ketogenic diets—rich in MCFAs—have gained interest for their potential to improve mitochondrial energy metabolism. A case study by Phillips *et al*. reported improved functional scores, respiratory parameters, and quality of life in an ALS patient after 18 months on a time-restricted ketogenic diet. Although based on a single case, the results highlight the feasibility and possible neuroprotective impact of FA-based metabolic interventions in ALS (172). Furthermore, supplementation of a Mediterranean diet with coconut oil was shown to elevate plasma ketone body levels in ALS patients, suggesting that MCFAs may serve as alternative energy substrates that support motor neuron survival (173).

Collectively, these clinical findings highlight the therapeutic potential of FA-based interventions—particularly through modulating neuroinflammation and mitochondrial energy metabolism—in NDDs such as AD and ALS. However, challenges remain regarding formulation stability, effective brain delivery, and interindividual variability in therapeutic response. To advance clinical translation, future efforts should focus on structural optimization of FA molecules, development of targeted delivery systems capable of crossing the BBB, and precise patient stratification. These strategies may help shift FA therapies from nutritional supplementation toward evidence-based, mechanism-driven precision interventions.

## Discussion

FA chain length and saturation dictate their roles in NDDs. SCFAs, as core microbial metabolites, modulate NDD progression through epigenetic regulation (47), BBB maintenance, oxidative stress mitigation (92, 106), and neuroinflammation suppression (50, 52, 73). However, their effects are bidirectional. In AD, SCFAs increase microglial ApoE, worsening Aβ deposition (201). While in PD, they trigger microglial activation and α-Syn aggregation (6). These opposing outcomes depend on concentration, pathological microenvironment, and disease stage (7, 201). MCFAs improve ALS motor dysfunction through rapid energy provision and motor deficit alleviation (84, 118, 150). Among LCFAs, n-3 PUFAs exert central neuroprotective functions by enhancing neuronal antioxidant capacity (86, 87, 137), shifting glial phenotypes toward anti-inflammatory states (114, 119, 121), and suppressing pathogenic protein aggregation or phosphorylation in AD models (75, 87), with intervention efficacy being strongly influenced by genetic background (116, 132). Conversely, saturated LCFAs and n-6 PUFAs predominantly exacerbate neuronal damage and BBB disruption through proinflammatory signaling and oxidative stress pathways (127). Notably, NDDs consistently exhibit characteristic FA metabolic network disturbances, including reduced SCFA levels, dysregulated n-3/n-6 ratios, and accumulated LPO end-products, which collectively provide novel therapeutic targets for NDD modulation.

Based on these insights, we propose several key directions for future research into the intricate relationship between FAs and NDDs. Given the variability in therapeutic responses based on genotype, disease stage, and microenvironment, tailored intervention strategies are crucial (174, 175, 176). This includes establishing personalized FA profiles through advanced metabolomics and multiomics approaches, enabling optimized dosing regimens and identification of responsive patient subpopulations. Second, in-depth exploration and targeted modulation of the gut microbiota-FA-gut-brain axis may yield breakthroughs in NDD therapy. Considering that gut pathology often precedes central neurodegeneration, profiling gut or plasma FA signatures could offer promising early biomarkers for timely intervention (40, 137). Clarifying the causal relationships between dysbiosis and FA metabolic disturbances, along with the development of specific probiotics, prebiotics, or FA derivatives, may provide novel, noninvasive treatment strategies. In particular, the mechanisms by which SCFAs regulate tight junction proteins and maintain CNS homeostasis remain to be fully elucidated. Third, optimizing delivery strategies—especially through nanotechnology and genetic engineering—could enhance the brain-targeting efficiency of FAs and their derivatives (177, 178). Finally, accelerating the translation of basic research into clinical applications is imperative. Special attention should be given to species differences (e.g., the disproportionate colonic structure and SCFA metabolism in rodents compared to humans), which may hinder translational relevance. There is also a pressing need to establish standardized methods for dynamic monitoring of gut and plasma FA profiles. Future clinical trials should be rigorously designed, including multi-ethnic cohorts, and adopt optimized recruitment strategies. Large-scale, randomized controlled trials (16, 41) are essential to validate the efficacy of FAs and their derivatives in human NDDs, as well as to explore combination therapies—such as coadministration with anti-inflammatory or antioxidant agents—to enhance therapeutic effectiveness.

In conclusion, FAs function as pivotal metabolic and signaling molecules regulating the key pathological pathways of NDDs. Continued research and clinical translation in this field may open new therapeutic avenues and offer effective strategies to improve the quality of life and clinical outcomes for the growing population affected by neurodegenerative disorders in an aging global society.

## Conflict of interest

The authors declare that they have no conflicts of interest with the contents of this article.
